# Urethrogluteal Fistula Developing Secondary to the Use of Clean Intermittent Self-Catheterization: First Case Report in the Literature

**DOI:** 10.1155/2014/218037

**Published:** 2014-10-02

**Authors:** Aliseydi Bozkurt, Mehmet Karabakan, Mehmet Soyturk, Erkan Hirik, Barış Nuhoglu

**Affiliations:** ^1^Urology Department, Erzincan University, Mengücek Gazi Training and Research Hospital, Basbaglar Mahallesi, 1430 Sokak, 24000 Erzincan, Turkey; ^2^Radiology Department, Erzincan University, Mengücek Gazi Training and Research Hospital, 24000 Erzincan, Turkey

## Abstract

Clean intermittent self-catheterization is the standard method for bladder evacuation in these patients today. The patient was diagnosed with urethrogluteal fistula and gluteal-perineal abscess by radiological evaluation. Gluteal drainage decreased after cystostomy. In our paper, a case of urethrogluteal fistula and pelvic urinoma that developed as a result of the use of clean intermittent self-catheterization (CISC), which is rarely found in the literature, is presented.

## 1. Introduction

Clean intermittent self-catheterization (CISC) is the preferred method for bladder emptying in patients with spinal cord injury.

It was first proposed by Guttmann in the form of sterile catheterization in 1966 and it was applied in the form of nonsterile catheterization by Lapides et al. in 1972 [1].

In several studies, it was reported to have preventive or therapeutic effects in the case of diseases such as hydronephrosis, vesicoureteral reflux, urinary tract infections, and urinary incontinence [2].

Although it has obvious advantages compared to continuous catheterization, a number of complications including urethral stricture and formation of urethral false passage may develop due to recurrent urethral catheterizations. In our case, diagnosis and treatment of urethrogluteal fistula and a very large pelvic urinoma that developed as a result of the use of CISC for emptying neurogenic bladder are discussed.

## 2. Case Report

A 36-year-old male patient was admitted to our clinic with complaints of pain, swelling, and redness that developed in suprapubic, scrotal, and gluteal areas and of difficulty in implementing the CISC, dysuria, and fever for the last one week. He has been paraplegic due to a spinal cord injury which took place 17 years ago by a firearm. He has been implementing CISC with single-use hydrophilic coated catheters for the last 5 years.

He is an obese, diabetic (type 2, controlled) patient poor in self-care and with normal mental functions. There was no history of urethral stricture or catheterization difficulties on his medical background. A painful, red, swollen lesion looking like an abscess which starts from suprapubic area, covers the entire scrotum and extends to left thigh and gluteal region was observed. In left gluteal region, a cutaneous fistula orifice with purulent discharge was observed. Biochemical and heamatologic analyses were normal.

**Figure 1:**
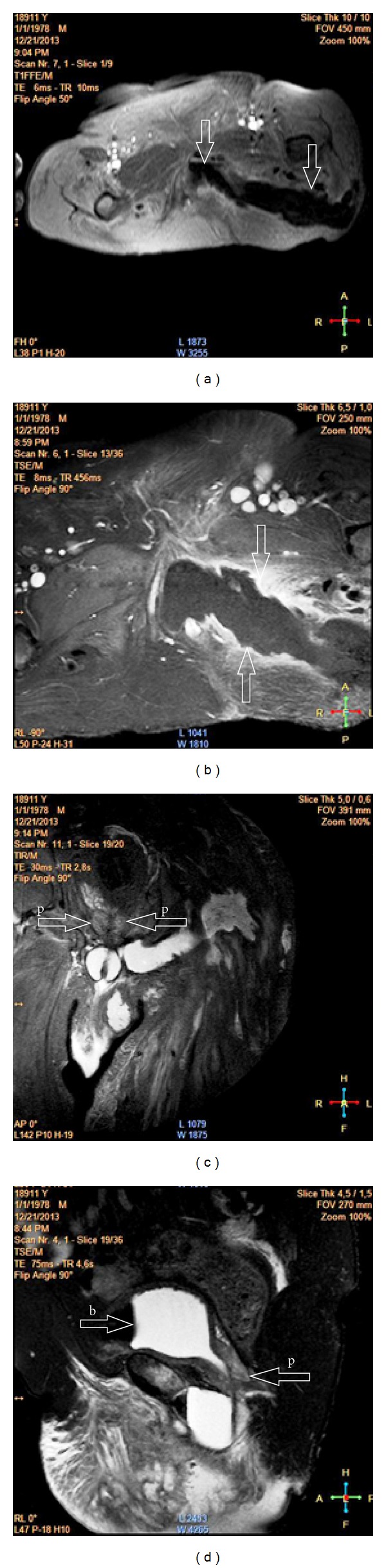
((a) + (b)) Fluid collection with dense peripheral contrast uptake in sections with contrast extending from deep pelvic muscle groups to the perineum and left gluteal region. ((c) + (d)) p: prostate, b: bladder. This collection extends to perineum, left scrotum, and left gluteal region and is connected to the skin from the gluteal region.

**Figure 2:**
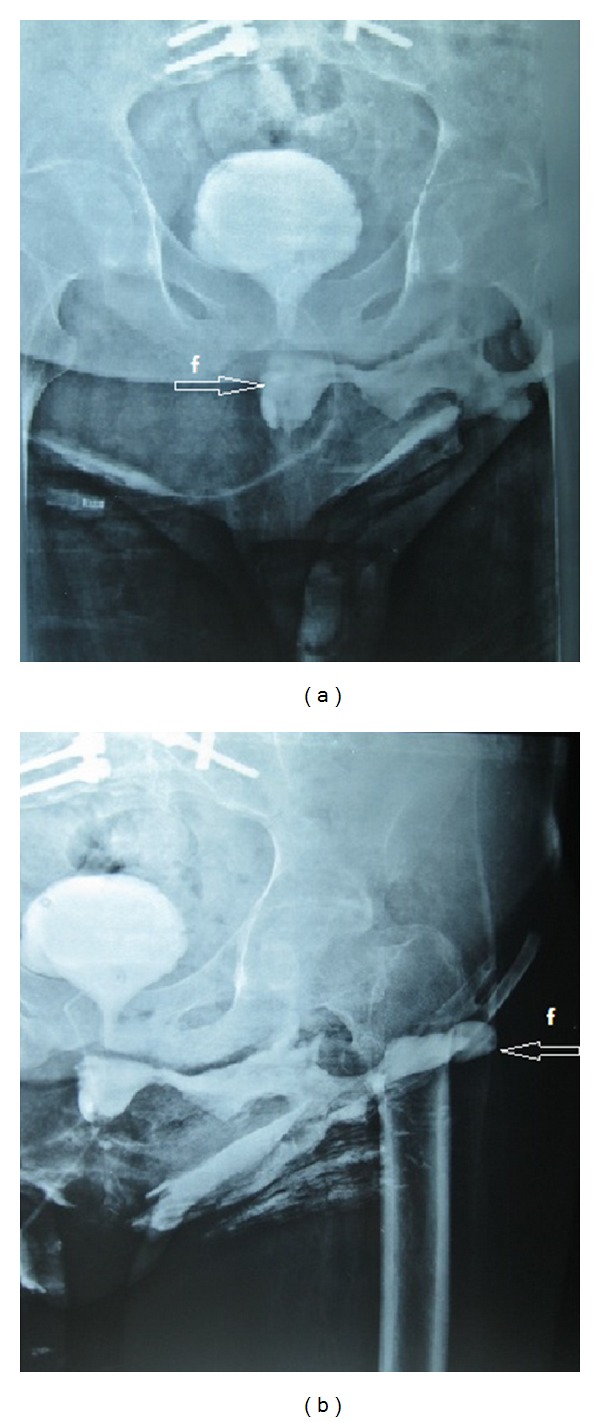
In cystourethrography, fistula tract and urinoma filled from a defect in the urethra were observed; f: fistula tract.

During superficial diagnostic ultrasonography (USG), a 21 × 5 cm collection of fluid extending from left gluteal region to the perineum was observed. Pelvic magnetic resonance imaging (MRI) revealed a large collection of fluid of about 22 × 5.5 cm with dense content and dense peripheral contrast uptake extending from left gluteal region to the pelvic floor (Figure 1). In cystourethrography of the patient, the contrast agent was seen moving from prostatic urethra toward the gluteal region (Figure 2).

Gluteal abscess drainage in the presence of USG was performed on the patient and percutaneous suprapubic cystostomy catheter was inserted. During his postcystostomy follow-ups, a reduction in gluteal drainage was observed.

After urinary tract infection of the patient was brought under control, cystoscopy was performed to confirm our diagnosis. In cystoscopy, a wide and deformed fistula tract extending to trigon base in the posterior urethra was seen. After cystoscopy, the patient was diagnosed with urethrogluteal fistula. In order to enable spontaneous closure of fistula tract, follow-up of the patient with suprapubic cystostomy catheter was recommended. At the third month follow-up visit, the drainage from fistula was minimal. Therefore, it was decided to continue the follow-up with suprapubic cystostomy.

## 3. Discussion

As a result of recurrent catheterization, a local urethral trauma occurs in male patients who implement CISC, which increases with long-term use of CISC [3, 4].

Incidence of formation of urethral false passage during the use of CISC was reported to be 3–9%, while that of urethral stricture was reported to be 1–9% in the literature [2]. Abscess formation as a complication of intermittent catheterization has only been described twice previously [5, 6].

In our patient, we encountered a complication induced by CISC, which was rarely reported in the literature previously. A fistula extending from the posterior urethra to the gluteal region was detected in our patient.

Imaging methods, including cystourethrography, cystourethroscopy, fistulography, and magnetic resonance imaging (MRI) can be used to diagnose fistula tract and detect possible factors that may cause it [7]. In our case, wide abscess foci extending from posterior urethra to gluteal muscle groups were observed in MRI. A large fistula tract extending between the urethra and the gluteal region was revealed by cystourethrography. Wide urethral defect was seen in the posterior urethra during cystourethroscopy performed on the patient.

Michielsen and Wyndaele [8] detected a false passage that developed as a result of traumatic catheterization of external sphincter area in one of their patients and thought that this was caused by sphincter spasticity. Similarly, large fistula tract arising from immediate proximal of external sphincter was thought to be due to sphincter spasticity in our patient.

Detailed information on recovery of fistulas extending from urethra toward muscle groups is not available in the literature. During the first treatment, it is aimed to prevent urinary sepsis using appropriate antibiotics [8]. Some of the fistulas can be treated with simple procedures such as urethral catheterization and follow-up [6, 7]. We diagnosed our patient with fistula and performed his follow-up by suprapubic catheterization. Suprapubic gluteal abscess foci was seen to recover during catheterization; however, complete closure of the fistula tract could not be achieved. In complicated cases, primary repair techniques for fistula as well as graft methods can be utilized [9]. Koleilat et al. [10] achieved complete recovery by urethral catheterization and follow-up in 7 of 9 patients with urethral false passage, while they implemented urinary diversion in one of the patients who did not recover. In our case, it is planned to carry out a primary urethral repair if complete recovery cannot be achieved by follow-up with suprapubic catheterization. Urinary diversion is also among the options if urethral defect does not close.

## 4. Conclusion

The CISC-induced urethral fistulas can, albeit occasionally, be seen. Treating patients with a temporary urethral stent and antibiotics seem to be a good treatment option. In the case of fistulas which do not recover despite this, primary repair, the use of graft, and urinary diversion may be considered.
